# Suicidal ideation, attempt and associated factors among people with cancer attending cancer center, eastern Ethiopia

**DOI:** 10.3389/fpsyt.2023.1184921

**Published:** 2023-09-15

**Authors:** Kabtamu Nigussie, Dejene Tesfaye, Lemesa Abdisa, Lidiya Tolosa, Tilahun Bete, Kabtamu Gemechu, Abraham Negash, Addisu Sertsu, Jerman Dereje, Gebiso Roba Debele, Deribe Bekele Dechasa

**Affiliations:** ^1^Department of Psychiatry, School of Nursing and Midwifery, College of Health and Medical Sciences, Haramaya University, Harar, Ethiopia; ^2^Department of Nursing, School of Nursing and Midwifery, College of Health and Medical Sciences, Haramaya University, Harar, Ethiopia; ^3^School of Medical Laboratory Science, College of Health and Medical Sciences, Haramaya University, Harar, Ethiopia; ^4^Department of Midwifery, School of Nursing and Midwifery, College of Health and Medical Sciences, Haramaya University, Harar, Ethiopia; ^5^Department of Public Health, College of Health Science, Mettu University, Metu, Ethiopia

**Keywords:** magnitude, associated, cancer, suicidal ideation, attempt, eastern Ethiopia

## Abstract

**Background:**

Suicide is one of the most common public health problems and the second leading cause of death among individuals 15–29 years of age. Suicidal ideation and attempt are one of the common psychiatric emergence in patients with cancer that needs early detection and management before patients end their lives. Therefore, the aim of the study was to assess the magnitude of suicidal ideation, attempts, and associated factors among people with cancer in Eastern Ethiopia.

**Methods:**

An institutional-based cross-sectional study design was conducted among 362 participants. A composite international diagnostic interview was used to evaluate suicidal ideation and attempt. Epi-Data version 4.6.2 was used for data entry, and SPSS version 20 was used for analysis. Logistic regression analysis was done to identify associated factors for both suicidal ideation and attempt. *p*-values less than 0.05 are considered statistically significant, and the strength of the association will be represented by an adjusted odds ratio with a 95% confidence interval.

**Results:**

The magnitude of suicidal ideation and attempt among people with in this study was 22.9% [95% CI, 18.7–27.4] and 9.8% [95%CI, 6.7–12.8] respectively. Being living alone [AOR = 4.90, 95% CI, 2.08–11.90], and having depressive symptoms [AOR = 3.28, 95% CI, 1.37–7.73], female ([AOR = 1.53, 95% CI, 1.30–3.23], anxiety symptoms [AOR = 3.06, 95% CI, 1.35–6.73)] and having poor social support [AOR = 3.08, 95% CI, 1.72–5.05], were significantly associated suicidal ideation whereas, Being living alone [AOR = 2.89, 95% CI, 1.09–7.65], having a depressive symptoms [AOR = 4.88, 95% CI, 1.45–13.28], being divorced/widowed [AOR = 3.46, 95% CI, 1.09–10.09] and stage four cancer [AOR = 5.53, 95% CI 2.97–7.47] were significantly associated with suicidal attempt.

**Conclusion:**

Nearly one-quarter and one-tenth of people with cancer have suicide ideation and attempt, respectively. Suicidal behavior among cancer patients was found in this study to be a common problem. Living alone, having depressive and anxiety symptoms, being a female, having poor social support, and being in forth stage of cancer were risk factors for suicide. Therefore, early screening, diagnosing, and treating suicide and its factors will be mandatory and expected from health care providers and non-governmental organizations.

## Introduction

Suicide is the Latin word for “self-murder.” Self-inflicted death with either implicit or explicit evidence that the person planned to die implies the fatal act of a person’s deliberate and intentional destruction of one’s own life (1). Suicide, also described as a complex process, goes from suicidal ideation to planning, attempt, and a completed suicide. Some people take their life seemingly without planning for days, weeks, or even years on end, while others do so. Suicidal ideation can be active, where individuals have a specific plan and intent to die, or passive, where the patient feels that life is not worth living or that it would be better to be dead (2). An attempt at suicide is when someone makes a suicide attempt but manages to pull through and lives. It is self-harmful conduct with a non-lethal result accompanied by either implicit or explicit indicate that the person intended to die (3).

Suicide is the 10th leading cause of death for individuals around the world and one of the 2nd and 3rd leading causes of death for people 15–29 and 15–44 years of age, respectively (4, 5). There are one million suicide deaths annually or one every 40 s and accountable for 2.4% of the worldwide burden of illnesses (1). Every year, between 10 and 20 million people attempts suicide. While these attempts do not result in death, they do necessitate medical and mental health attention. Every year, another million people—family members and close friends—are impacted by suicide. According to World Health Organization (WHO) research, the annual and the lifetime of suicidal ideation was 2 and 9%, and suicidal attempt was 0.3, and 0.4%, respectively. Suicide deaths constitute more than 50% of all deaths from war and homicide combined, despite an undercount of the true number due to insufficient death registration and social denials about suicide. In the world, more than 800,000 individuals commit suicide each year, or one every 40 s (1, 4).

According to the WHO report, the global cancer burden is estimated to have increased by 18.1 million new cases and 9.6 million deaths in 2018. One out of five men and one out of six women worldwide develop cancer during their lifetime, and one in eight men and one in 11 women die from the disease. By 2030, cancer incidence and mortality in Africa will have two-folded to 1.28 million new cases and 970,000 deaths per year (6). By 2050, Ethiopia’s population of more than 100 million people would rise to the 9th -most populous country in the world, with a projected corresponding increase in the cancer burden (7, 8). Just 6.4% of cancer patients who have clinically significant major depressive disorder get identified and receive therapy, despite the fact that 50% of cancer patients do (9). Suicidal conduct is probably common among Ethiopian cancer patients given the significant link between untreated depression and suicidality (10–12). A study done in China among 517 cancer patients who are hospitalized by using a single-item questionnaire showed that the 1-month prevalence of suicidal ideation was 15.3% in Chinese cancer inpatients, and depression and anxiety symptoms, pain, last stage of cancer, poor performance status, surgical treatment, and poly-treatment were significantly associated with suicidality (13). Another study done in terminally ill Japanese cancer patients indicated that suicidal ideation was sub threshold in 26.4% of patients and present in 8.6% of them (14). Interest in requesting euthanasia was strong for 5.0% of patients, moderate for 2.9% of patients, and weak for 5.0% of patients (14).

The incidence of suicidal behavior among cancer patients is caused by different reasons, including the psychological stress caused by the diagnosis of cancer, long duration and side effect of treatment, multiple hospitalizations, disruption in life, decrease quality of life, and immunological disturbances (15). In addition, numerous studies have discovered that a recent diagnosis of central nervous system cancer (head, neck) and gender, age, social isolation, lack of social support, substance abuse, depression, and anxiety are all associated risk factors for both suicidal ideation and attempts in cancer patients (5). One in four cancer patients (25.24%) who were hospitalized at a Spanish cancer unit in a study conducted there had high ratings for suicide ideation. The key risk factors for suicidal thoughts and attempts are despair, hopelessness, personality disorder, low social support, retirement age, and those who have attempted suicide in the past (16).

Even though most of the research on suicidal ideation, attempts, and completed among cancer patients has been conducted in developed countries, little attention is given to it outside of mental health settings in low and middle-income (LMI) countries. The presence of suicidal ideation with cancer produces complications with the treatment of cancer that lead to poor adherence with treatment, worsening of the condition, and affect quality of life. It also tends to make people unknowingly end their lives and increases mortality from the diseases. Different studies in Ethiopia indicated that the high magnitude of suicide was associated with severe mental illness and other chronic conditions like HIV, epilepsy, DM, HTN, and TB. But attention is not given to identify suicidality among cancer patients, which is a common problem in Ethiopia, and there is little study in Africa and no published study on this topic in eastern Ethiopia.

Therefore, this study was intended to fill this gap by assessing the prevalence and associated factors of suicidal ideation and attempt among people with cancer and showing the scope of the problem in the study areas that are paramount for supportive care to integrate mental health care in cancer management. Furthermore, the result of this study was to provide information for health professionals to design appropriate solutions for the problem and to integrate psychiatric services into the treatment of cancer.

## Methods and materials

### Study area, design, period, population, and eligibility

An institution-based cross-sectional study design was conducted in the eastern part of Ethiopia at the Cancer Center located in Harar town. Harar Town is the capital city of Harari Regional State, one of the 11 states in Ethiopia, and it has a total population of 259,260, of which 130,097 are female. It is located 526 kilometers to the southeast of Addis Ababa and has 9 woredas and 36 kebeles’ (17 rural and 19 urban) with a total of 59,487 households. It has an estimated area of 333.94 square kilometers and an estimated population density of 595.9 people per square mile (17). Hiwot Fana specialized university hospital (HFSUH) delivers wider health care services to approximately 5.2 million people in the catchment area. HFSUH is a teaching hospital of Haramaya University, and it has 235 beds and 410 health professionals. HFSUH has different service areas, including chronic disease OPD, emergency OPD, medical, surgical, pediatrics, psychiatry, gynecology, and obstetrics wards, cancer, and ICUs. The eastern part of the cancer center was located in HFSUH. The study was carried out from April 1, 2021, to May 15, 2022. All patients with cancer who visit at Hiwot Fana specialized university hospital cancer center and available during data collection were, respectively, source of the population and study population. All people (age greater than or equal to 18 years) who had been clinically diagnosed with cancer and had an appointment at the out-patient clinic at Hiwot Fana Comprehensive specialized university hospital (HFSCUH) were included in this study, whereas patients who were unable to communicate and seriously ill at the time of the data collection period were excluded.

### Sample size determination and sampling procedure

By using the following assumptions, the minimum sample size required for the study was calculated by using the single population proportion formula,n=Zα22P1−Pd2. Where *n* = minimum sample size required for the study, *Z* = standard normal distribution (Z = 1.96) with a confidence interval of 95% and α = 0.05. P = For suicidal attempt, the magnitude of suicidal ideation among cancer patients was 8.4% (18) with *d* = 0.03 tolerable margin of error

$n=\;\frac{{\left(1.96\right)}^{2}\;\times \;0.084\;\left(1-0.084\right)}{0.03^{2}}=329.$

Then, adding 10% (329 × 0.1 = 32.9 ≈ 33) of non-respondents, the total sample size for this study is 329 + 33 = 362. Therefore, 362 were used as the sample size for this study. A systematic random sampling technique was used with a calculated *K*-value of 2. The first study participant was selected by a lottery method, and the next study participants were chosen at a regular interval (every 2) and interviewed by data collectors.

### Data collection instruments and data collectors

The study questionnaire has six components. Socio-economic characteristics of participant were collected by structured socio-economic questionnaires; clinical related factors and substance related were collected by structured questionnaires. A dependent variables, suicidal ideation and attempt, were assessed by items that are adapted from a module of the World Mental Health (WMH) Survey Initiative Version of the World Health organization, the Composite International Diagnostic Interview (CIDI), in which suicide was studied and validated in Ethiopia both at clinical and community settings with internal consistency Cronbach’s alpha =0.97 (19, 20). The internal consistence, Cronbach’s alpha of CIDI in the current study was 0.93.

Social support was collected using the Oslo-3 item social support scale, which is a 3-item questionnaire commonly used to assess social support and has been used in several studies. The sum score scale ranged from 3 to 14, and had 3 categories: poor support 3–8, moderate support 9–11, and strong support 12–14 (21). The internal consistence, Cronbach’s alpha of Olso-3 items in the current study was 0.82.

Depression and anxiety were collected by the Hospital Anxiety Depression Scale (HADS), which was validated in Ethiopia with internal consistency of 0.78 for anxiety, 0.76 for the depression subscale, and 0.87 for the full HADS scale. It has 14 item questioners commonly used to screen for symptoms of anxiety and depression that are separated into two parts, which is a 7-item sub-scale for anxiety and depression. The items are rated on a four-point Likert scale ranging from 0 to 3, giving a maximum and minimum score of 0 and 21, respectively. If the respondent’s score was 8 or higher, this indicates anxiety and depression (21). The internal consistence, Cronbach’s alpha of HADS in the current study was 0.87 and 0.85 foe depression and Anxiety, respectively. Pain will be assessed by using visual analog scale (VAS) which has 0–100 mm range individual who has 0-4 mm considered as no pain 5–44 mild pain, 45–74 moderate and >75 sever pain (22).

Substance-related factors were assessed by the Alcohol, Smoking, and Substance Involvement Screening Tool (ASSIT), which is a brief screening questionnaire developed by the WHO to find out about people’s use of psychoactive substances. This was used to assess the subject’s current and past substance use history, and data was collected by three trained health professionals with a bachelor of science degree and supervised by one health professional who has a master of science, degree (23). The internal consistence, Cronbach’s alpha of ASSIT items in the current study was 0.83.

### Operational definitions

#### Suicidal ideation

If the respondent answers yes to the question, “Have you seriously thought about committing suicide within the last month?” then the respondent has suicidal ideation according to the suicide module of the CIDI (20).

#### Suicidal attempt

If the respondent answers yes to the question “Have you attempted suicide in the last month?” the respondent has a suicidal attempt from the CIDI’s suicidality module (20).

#### Social support

According to the Oslo-3 social support scale, which ranges from 3 to 14, those respondents who score 3–8 are considered to have poor social support, those who score 9–11 are considered to have moderate social support, and those who score 12–14 are considered to have strong social support (21).

#### Hospital anxiety and depression scale

The hospital anxiety depression scale (HADS) was a 14-item questionnaire commonly used to screen for symptoms of anxiety and depression. It was separated into two parts, each with a 7-item subscale for anxiety and depression. The items are rated on a four-point Likert scale ranging from 0 to 3, giving a maximum and minimum score of 0 and 21, respectively. If the respondent’s score of 8 or above indicated anxiety and depression (21)

#### Pain

According to visual analog scale (VAS) which has 0–100 mm range, those who score 0–4 mm no pain, 5–74 mm and 75–100 have pain (22).

#### Current use

Using at least one of a specific substance for a non-medical purpose within the last 3 months, according to the Alcohol, Smoking, and Substance Involvement Screening Tool (ASSIT) (23).

#### Ever use

Using at least one of any specific substance for the nonmedical purpose at least once in a lifetime according to ASSIST (23).

### Study variables

#### Dependent variable

Suicidal ideation (yes/no)

Suicidal attempt (yes/no)

#### Independent variables

Socio-demographic variables; sex, age in years, marital status, living arrangement, religion, occupational status, educational status, residence, clinical factors; family history of mental illness, depressive symptoms, anxiety symptoms, stage of cancer, types of treatment, family history of suicide, family history of mental illness, duration since diagnosed, treatment side effect, pain, and comorbid medical illness, substance-related factors; current and lifetime substance use, and psychosocial factor; social support.

### Data quality control

Data will be collected through a face-to-face interview using an Amharic or Afan Oromo version of a pre-tested questionnaire and by reviewing the patient’s chart using a check list prepared in English. The structured questionnaires were translated into Amharic and Afan Oromo, which almost all participants can understand. To check its consistency with its English version, it was retranslated to the original version. All structured questionnaires were pre-tested at a nearby hospital before 1 week of exact data collection among 5%(18) of the total sample size.

### Data processing and analysis

Coded and checked data was entered into the computer using EPI Info version 4.6.2 and imported to the Statistical Package for Social Science (SPSS) window software version 20. Bivariate and multivariate binary logistic regression analysis was conducted to determine the presence of a statistically significant association between independent and dependent variables. Those variables that had a value of *p* of less than 0.25 in the binary logistic model were taken to the multivariable logistic model. *p*-values less than 0.05 were considered statistically significant with outcome variables, and the strength of the association was presented by the odds ratio with a 95% confidence interval (CI). Hosmer–Lemshow goodness and maximum likelihood were checked for mode fitness variance inflation factors (VIF) used to check relations between variables.

### Ethical consideration

Ethical clearance was obtained from the Institutional Health Research Ethics Review Committee of the College of Health and Medical Sciences at Haramaya University. The confidentiality of respondents was maintained by letting them fill out the questionnaire anonymously. Completed questionnaires and computer data were kept confidential by password security. Informed, voluntary, written, and signed consent was taken from each respondent. The respondent was aware of their right to withdraw from the interview at any time they wished. Respondent were assured that if they wished to refuse to participate, their care or dignity was not compromised in any way to which they were otherwise entitled. Appropriate COVID-19 prevention protocol was followed throughout the research process. Respondent with severe suicidal ideation or attempts were linked to the psychiatric department of the hospital for further evaluation and treatment.

## Results

### Socio-economic characteristics of study participants

From a total of 362 samples, 358 participants were provided consent and included in the study, with a response rate of 98.9%. The median age of respondents was 35 years; the interquartile range (IQR) was 29–47 years. More than half, 59.2, and 55.2%, of the study participants were male and married, respectively. Regarding the living conditions of the study respondents, more than two thirds (69.0) were living with family, and around half of them (52.4%) were orthodox by religion, as shown below in (Table 1).

**Table 1:** Socio-economic characteristics of people with cancer, Eastern, Ethiopia, 2022 (*n* = 358).

Variables	Frequency (*n* = 358)	Percentage
Sex		
Male Female	212 146	59.2 40.8
Age		
18–24 25–31 32–38 39–45 >45	108 125 49 35 41	30.2 34.9 13.7 9.8 11.5
Marital status		
Married Single Divorced/widowed	198 106 54	55.3 29.6 15.1
Living condition		
With family Alone	247 111	69.0 31.0
Religion		
Orthodox Muslim Protestant Others	189 104 52 13	52.4 29.5 14.5 3.6
Occupation		
Government worker Merchant Framer Student Household worker Unemployed	28 76 117 41 37 59	7.8 21.2 32.7 11.5 10.3 16.5
Education		
No formal education Primary school Secondary school Diploma and above	79 159 83 37	22.1 44.4 23.2 10.3
Residence		
Rural Urban	214 144	59.8 40.2

### Clinical factors of respondents

The majority, 69.6 percent of respondents, reported the time since diagnosis as 18 months and above. More than one fourth, 27.2%, and nearly one third, 32.1%, of the study participants had breast cancer and pain, respectively, and around half of the participants, 57.8%, had used chemotherapy-type treatment. Out of the total study participants, 43.6 and 29.3% were found to have depression and anxiety symptoms, respectively, whereas 9.8% had comorbid other medical illnesses. Regarding substance use, 40.2% of participants had ever used substances, and 25.1% were currently using substances, but only 20.7% of study participants had strong social networks, as shown below in Table 2.

**Table 2:** Description of clinical, psychosocial and substance use characteristics of people with cancer, Eastern, Ethiopia, 2022 (*n* = 358).

Category	Frequency (*n* = 358)	Percentage
Time since diagnosis		
<18 month ≥18 months	109 249	30.4 69.6
Taking other medication		
Yes No	53 305	14.8 85.2
Anatomical site of cancer		
Breast Genitourinary GI Gynecological Hematological HNC Lung Others *	98 12 47 52 60 22 41 26	27.2 3.4 13.1 14.5 16.8 6.1 11.6 7.8
Presence pain		
Yes No	115 243	32.1 67.9
Types of treatment		
Chemotherapy Surgery Combined	207 77 74	57.8 21.5 20.7
Stage of cancer		
Stage 1 Stage 2 Stage 3 Stage 4	55 98 155 50	15.4 27.4 43.3 14.0
Depressive symptoms		
Yes No	156 202	43.6 56.4
Anxiety symptoms		
Yes No	105 253	29.3 70.7
Comorbid other medical illness		
Yes No	35 323	9.8 90.2
Family history of suicidal attempt		
Yes No	44 313	12.3 87.7
Family history of committed suicide		
Yes No	31 327	8.7 91.3
Family history of mental illness		
Yes No	36 321	10.1 89.9
Family history of cancer		
Yes No	31 327	8.7 91.3
Treatment side effect		
Yes No	57 301	15.9 84.1
Social support		
Poor Moderate Strong	118 166 74	33.0 46.4 20.7
Ever substance use		
Yes No	144 214	40.2 59.8
Current substance use		
Yes No	90 268	25.1 74.9

Others *, pancreatic cancer, skin cancer, sarcoma and liver cancer.

### Factors associated with suicidal ideation among people with cancer, eastern Ethiopia

In bivariate logistic regression analysis variables like being female, single, divorced, widowed, living alone, primary school educational level, having depressive symptom, having anxiety symptoms, current and ever substance use and poor social support were significantly associated with suicidal ideation. However, in the multivariate logistic regression analysis variables like a female, ling alone, anxiety symptoms, depressive symptoms and poor social support were statistically significantly associated with suicidal ideation with a value of *p* less than 0.05.

In this study, the odds of having suicidal ideation among respondents with were female were about 1.53 times higher as compared to participants those being male [AOR = 1.53 95% CI, 1.30–3.23] and the odds of having suicidal ideation among participants who living alone were 4.90, times higher as compared to respondents who were live with their family [AOR = 4.90 95% CI, 2.08–11.90].

The odds of having suicidal ideation among respondents who had an anxiety symptoms and depressive symptoms were 3.06 and 3.28 times higher as compared to respondents who had no anxiety symptoms and depressive symptoms [AOR = 3.06, 95% CI, 1.35–6.73] and [AOR = 3.28, 95% CI, 1.37–7.73], respectively. Besides, the odds of having suicidal ideation among participants who had poor social support was 3.08 times higher as compared to respondents who had strong social support [AOR = 3.08, 95% CI, 1.72–5.05] as shown in (Table 3).

**Table 3:** Bivariate and multivariate logistic regression analysis between some selected factors and suicidal ideation among people with cancer, Eastern, Ethiopia, 2022.

Explanatory variables	Suicide ideation		COR (95%CI)	AOR (95%CI)
	Yes	No		
Sex				
Male Female	38 44	174 102	1 1.98 (1.20–3.25)	1 1.53 (1.30–3.23) *
Marital status				
Married Single Divorced/widowed	21 32 29	177 74 25	1 3.65 (1.97–6.73) 9.78 (4.85–19.67)	1 0.75 (0.58–5.29) 1.66 (0.51–5.46)
Living condition				
With family Alone	26 56	221 35	1 8.65 (4.99–15.02)	1 4.90 (2.08–11.90) **
Occupational status				
Governmental Merchant Framer Student House wife Unemployed	4 10 17 7 11 33	24 66 100 34 26 26	1 0.91 (0.26–3.17) 1.02 (0.31–3.31) 1.24 (0.33–4.69) 2.54 (0.74–9.06) 7.62 (2.33–24.70)	1 0.35 (0.02–7.88) 0.32 (0.14–7.36) 0.17 (0.06–4.52) 0.47 (0.02–13.09) 0.52 (0.22–11.82)
Educational status				
No formal education Primary school Secondary school Diploma and above	26 43 15 4	59 116 68 33	2.79 (0.88–8.88) 3.06 (1.02–9.14) 1.82 (0.56–5.92) 1	4.48 (0.27–7.85) 2.52 (0.30–6.93) 1.38 (0.09–2.47) 1
Anxiety symptoms				
Yes No	47 35	58 218	5.05 (2.99–8.53) 1	3.06 (1.35–6.73) * 1
Depression symptoms				
Yes No	66 16	90 186	8.52 (4.67–15.56) 1	3.28 (1.37–7.73) * 1
Ever substance use				
Yes No	51 31	93 183	3.24 (1.94–5.39) 1	2.21 (0.73–4.53) 1
Current substance use				
Yes No	34 48	56 220	2.78 (1.64–4.72) 1	0.79, (0.24–2.63) 1
Social support				
Poor Moderate Strong	57 12 13	61 154 61	4.39 (2.18–8.82) 0.37 (0.16–0.85) 1	3.08 (1.72–5.05)* 0.52 (0.16–1.68) 1

*p < 0.05, and **p < 0.001; Chi square = 9.07, DF = 8, Hosmer–Lemshow test = 0.83.

### Factors associated with suicidal attempt among people with cancer, eastern Ethiopia

In bivariate logistic regression analysis variables like being divorced or widowed, living alone, having other comorbid medical illness,4th stage of cancer, depressive symptoms, poor social support, current and ever substance use were significantly associated with suicidal attempt. However, in the multivariate logistic regression analysis variables like a divorced or widowed, living alone, depressive symptoms and 4th stage of cancer were statistically significantly associated with suicidal attempt with a value of *p* less than 0.05.

In this study, the odds of having suicidal attempt among respondents with were living alone were about 2.89 times higher as compared to participants those were lived with family [AOR = 2.89 95% CI, 1.09–7.65], and the odds of having suicidal attempt among participants who were widowed/divorced were 3.46 times higher as compared to respondents who were married with [AOR = 3.46 95% CI, 1.09–10.09].

The odds of having suicidal attempt among respondents who had a depressive symptoms were 4.88 times higher as compared to respondents who had no depressive symptoms [AOR = 4.88, 95% CI, 1.45–13.28] and odds of having suicidal attempt among participants who were at 4^th^ stage of cancer was 5.53 times higher as compared to participants who were at first stage of cancer [AOR = 5.53 95% CI, 2.97–7.47] as shown in Table 4.

**Table 4:** Bivariate and multivariate logistic regression analysis showing an association between factors and suicidal attempt among people with cancer, Eastern, Ethiopia, 2022.

Variables	Suicidal attempt		COR (95%CI)	AOR (95%CI)
	Yes	No		
Living arrangement				
With family Alone	11 24	236 87	1 5.92 (2.78–12.59)	1 2.89 (1.09–7.65) *
Marital status				
Married single Divorced/widowed	11 10 14	187 96 40	1 1.77 (0.73–4.32) 5.95 (2.52–14.06)	1 2.38 (0.73–7.81) 3.46 (1.09–10.09) *
Comorbid illness				
Yes No	10 25	25 298	4.77 (2.06–11.04) 1	1.84 (0.55–6.23) 1
Stage of cancer				
Stage 1 Stage 2 Stage 3 Stage 4	3 5 13 14	52 93 142 36	1 0.93 (0.21–4.06) 1.59 (0.44–5.79) 6.74 (1.80–20.06)	1 2.63 (0.52–3.49) 2.13 (0.32–6.52) 5.53 (2.97–7.47) **
Depression symptoms				
Yes No	31 4	125 198	12.25 (4.23–23.08) 1	4.88 (1.45–13.28) * 1
Ever substance use				
Yes No	28 7	116 207	7.14 (3.02–16.85) 1	1.89 (0.49–7.26) 1
Current substance use				
Yes No	20 15	70 253	4.82 (2.35–9.89) 1	1.76 (0.53–5.91) 1
Social support				
Poor Moderate Strong	25 3 7	93 163 67	2.57 (1.05–6.29) 0.18 (0.04–1.70) 1	1.05 (0.34–3.23) 0.13 (0.03–1.56) 1

*p < 0.05, and **p < 0.001, Chi square = 5.04, DF = 8, Hosmer–Lemshow test = 0.91.

## Discussion

The aim of this study was to assess the magnitude and associated factors of suicidal ideation and attempt among cancer patients. The magnitude of suicidal ideation and attempt was found to be 22.9% at 95% CI [18.7–27.4] and 9.8% at 95% CI [6.7–12.8], respectively. The magnitude of suicidal ideation in this study was 22.9%, which was in line with Spain’s 25.5% (16), Korea’s 20.1% (24), South Korea’s 24.7% (25), Italy 20% (26). However, the findings of this study are higher than those of previous studies conducted in Ethiopia Gondar 16.6% (27), China 15.3% (28), Korea 17.7% (29), Spain 11.7% (30), Canada 9.6% (31), the United States 12.4% (32), and Turkey 7.8% (33). The possible reason for the variation might be related to the difference in the duration of the study. For example, this study measured the lifetime magnitude of suicidal ideation, whereas the China study measured its one-month duration. The other reason might be the difference in the study area; the current study was a hospital-based study, whereas the Korea study was a community-based study among cancer patients. This might be because patients who are diagnosed and living in the community might not be in severe pain compared to those who have a follow-up because they might not want to end their life (27).

However, the finding of suicidal ideation in this study is lower than the study done in Ethiopia Mekelle 27.9% (18), South Africa 71.4% (34), and Portugal 34.5% (35). The inclusion criteria of participants could be the source of the variation. The Mekelle study includes all patients, whereas this study includes only those who have outpatient follow-up and excludes severely and acutely ill patients. These, in turn, will cause severely and acutely ill patients to have more thoughts of wanting to kill themselves than their counterparts. The South Africa study participants were only among those with cervix cancer, whereas this study includes all types of cancer.

The magnitude of suicide attempts in this study, 9.8%, was in line with Ethiopia Mekelle’s 8.9% (18), Turkey’s 12.74% (36), and Korea’s 12.7% (25), but higher than the study conducted in Ethiopia Gondar 5.5% (27), Turkey’s 4.2% (37), Colombia’s 4.5% (38), Sweden’s 1.07% (39), Canada 0.4% (40). The possible justification for these might be the difference in the study design, study participants, and sociocultural differences across countries.

However, the finding in this study was lower than China’s 14.6% (41). The main reason for this difference might be participant differences. The China study participants were older, and they are at an advanced stage of cancer. These factors can make people feel hopeless and increase the likelihood of attempting suicide later in life rather than earlier. The other reason could be the time difference. Currently, due to better awareness and communication, they get early diagnosis and treatment. These may in turn reduce the reaction to a cancer diagnosis and increase their coping ability.

Regarding the associated factor, being female in sex was significantly associated with suicidal ideation among cancer patients. The finding is consistent with Ethiopian studies conducted in Gondar (27) and Mekelle (18). The implication for this might be that women have different coping mechanisms and stressors. When females are compared to males, females have additional psychosocial stressors such as physical and sexual violence that disturb their emotions, and this feeling may cause the thought of ending one’s life (42). The other justification might be that, according to socialization theory, women express their worries through rumination that leads them to end their lives (43).

The finding of this study shows that a depressive symptom was found to be an independent predictor of suicidal ideation and attempt among cancer patients. Participants who have comorbid depressive symptoms were 3.28 and 4.88 times more likely to have suicidal ideation and attempt, respectively, compared with their counterparts. This finding agrees with studies of Ethiopia Gondar (27), Mekelle (18) Spain (16), China (28), Korea (24), Turkey (33). The implication for this might be that depression causes negative beliefs—such as negative beliefs about themselves, the world, and their environment—that lead them to end their lives. Another possible reason might be that depressed individuals have a loss of interest and feel hopeless, which is a cardinal feature that leads them to attempt suicide. Furthermore, depression may impair their decision-making capacity, which leads them to seek escape mechanisms from their stressful encounters (44–46).

The odds of having suicidal ideation were more than three times more likely to occur among participants who had a comorbid anxiety symptom than those who had no anxiety symptoms. The finding is consistent with the finding of Ethiopia Mekelle (18), China (28), Korea (24). Anxiety symptoms are explained by an excessive feeling of worries and anxiety that is beyond the individual’s control. It overwhelms the thoughts of the attacked individual and can lead individuals to suicidal ideation (47).

The odds of having suicidal ideation and attempts were nearly five and three times more likely to occur among participants who were living alone, respectively, than among those who were living with their family members, this finding is congruent (48). Evidence suggests that individuals who are living alone are isolated from their partners and family members. When they are ill or infirm, they have difficulty receiving assistance, which causes them to face serious social problems. It turns this factor risky, leading them to have suicidal ideation and attempt (49, 50).

The finding of this study shows that stage-four cancer was found to be a significant predictor for suicidal attempts among cancer patients. The finding is consistent with other studies in Ethiopia, Gondar (27), Mekelle (18), Korea (24), and the USA (36). The implication for this might be that at the fourth stage of cancer, the illness becomes more severe, and people suffer from the illness. At this time, the illness starts to distress individuals and causes them to lose hope, which leads them to kill themselves (51).

In the final model of suicidal attempts, being divorced or widowed and having poor social support was significantly associated with suicidal attempts. The result is similar in the studies from Ethiopia’s Gondar (27) and Mekelle (18). The justification for this might be that an individual who is divorced or widowed loses their primary support system of social, emotional, and material support, which results in an increased feeling of loneliness and social isolation. At the end, this will be attributed to depression. Again, this led them to commit suicide (52).

### Strengths and limitations of the study

Using a sufficient sample to represent the target population and using standard and validated tools to assess both dependent and independent variables was taken as the strength of the study. However, because this study is a cross-sectional study design, it cannot allow establishing a temporal relationship between the outcome and the independent variable. Another possible limitation of the study is the absence of information concerning factors such as the study population’s access to care and financial burden.

## Conclusion

Nearly one-quarter and one-tenth of cancer patients have suicide ideation and attempt, respectively. Suicidal behavior among cancer patients was found in this study to be a common problem. Living alone, having depressive and anxiety symptoms, being a female, having poor social support, and being in an advanced stage of cancer were contributing factors to suicide. Therefore, early screening, diagnosing, and treating suicide and its factors will be mandatory and expected from health care providers and non-governmental organizations. We also recommend for researchers to assess accessibility to care and financial burden of study participants.

## Data availability statement

The original contributions presented in the study are included in the article/supplementary material, further inquiries can be directed to the corresponding author.

## Ethics statement

The studies involving human participants were reviewed and approved by Ethical clearance was obtained from the Institutional Health Research Ethics Review Committee of the College of Health and Medical Sciences at Haramaya University. The patients/participants provided their written informed consent to participate in this study.

## Author contributions

KN was involved from inception to the design, acquisition of data, analysis, and interpretation, and drafting and editing of the manuscript. AS, AN, DT, LT, DD, LA, JD, KG, and TB were the co-authors who participated in the review of the article, tool evaluation, interpretation, and critical review of the draft manuscript. All authors have read and approved the final manuscript.

## Abbreviations

CIDI: Composite International Diagnostic Interview
DSM: Diagnostic Statistical Manual
HNC: Head and Neck Cancer
MINI: Mini International Neuropsychiatric Interview
WHO: World Health Organization
